# Small grain production as an adaptive strategy to climate change in Mangwe District, Matabeleland South in Zimbabwe

**DOI:** 10.4102/jamba.v11i1.652

**Published:** 2019-10-23

**Authors:** Tapiwa Muzerengi, Happy M. Tirivangasi

**Affiliations:** 1 Department of Community Development, University of KwaZulu-Natal, Durban, South Africa; 2 Department of Sociology and Anthropology, University of Limpopo, Sovenga, South Africa

**Keywords:** climate change, food security, accessibility, availability, utilisation, stability

## Abstract

This article assesses the feasibility of small grains as an adaptive strategy to climate change in the Mangwe District in Zimbabwe. The change in climate has drastically affected rainfall patterns across the globe and in Zimbabwe in particular. Continuous prevalence of droughts in Zimbabwe, coupled with other economic calamities facing the Southern African country, has contributed to a larger extent to the reduction in grain production among communal farmers, most of whom are in semi-arid areas. This has caused a sudden increase in food shortages, particularly in the Mangwe District, as a result of erratic rainfall, which has negatively affected subsistence farming. This article was deeply rooted in qualitative research methodologies. Purposive sampling was used to sample the population. The researchers used key informant interviews, focus group discussions and secondary data to collect data. Data were analysed using INVIVO software, a data analysis tool that brings out themes. The results of the study are presented in the form of themes. The study established that small grains contributed significantly to addressing food shortages in the Mangwe District. The study results revealed that small grains were a reliable adaptive strategy to climate change as they increased food availability, accessibility, utilisation and stability. Despite the significant contribution of small grains to addressing food shortages, there is a need for the government to come up with a vibrant small grains policy, and government support that is visible as well as market creation for small grains. The study further recommends that small grains in semi-arid areas can be a panacea to food insecurity in Zimbabwe.

## Introduction and background

Grappling with hunger and food insecurity is one of the major challenges that global communities, and Zimbabwe in particular, have been experiencing since the 1990s. In 2016, Zimbabwe declared a state of emergency as drought caused crop failures across the country, rendering many communities vulnerable and food-insecure (Zimbabwe Independent 2016; Tirivangasi 2018). This resulted in approximately 2.5 million people or more than a quarter of the population requiring food aid (Buchanan 2016; Zimbabwe Independent 2016). Mandisvika, Chirisa and Bandauko (2015) concurred with the findings of Chirimuuta and Mapolisa (2011) that 80% of Zimbabwe’s total land is made up of fertile agricultural land, yet the country struggles to be food secure. The food insecurity is attributed to many factors, including political and socio-economic factors; however, the most gruesome are the effects of climate change. The *Zimbabwe Human Development Report* (2017) notes that Zimbabwe’s staple food is very sensitive to temperature and precipitation changes, hence affecting production. Zimbabwe’s farmers grow maize, millet, sorghum and wheat to ensure food security. However, maize takes up approximately 80% – 90% of production (Zimbabwe Human Development Report 2017). This renders the country vulnerable to climate variation as a result of the maize crop’s sensitivities to climate change.

Smallholder farmers in the Mangwe District suffer from low incomes and standards of living, as well as poor nutrition, housing and health. This is aggravated by the fact that there is usually very little rainfall in the Mangwe District. Annual rainfall is less than 500 mm per year in this region (Shumba 2001). In this area, rain-fed agriculture fails 4 years out of 5 (Gukurume 2013). Thus, those who rely on rainfall in this area are still impoverished and they are faced with food insecurity. Gukurume (2013) argues that the absence of small grain crops in the Mangwe District further exacerbates food insecurity. As a result of these erratic rains, crop production on dry land has been low as compared to those farmers on irrigation schemes. Dry land farmers are limited to these low production crops such as millet, sorghum and maize (short season variety) because of inadequate rains in these regions. The discourse around food (in)security issues evolved in parallel with the definition of food security itself and shows changes in the areas that policy should prioritise, often informed by new unfolding food (in)security threats. In recent years, the sudden rise in food prices, the excrescent scarcity of inputs such as land and water, exclusion of women coupled with a lack of political will and with the sprouting of new food-related calamities, such as obesity, have required a complete revisit of the strategies to achieve food security. It is against this background that this study seeks to examine the impact of small grains as an adaptive strategy to climate change.

The Food and Agriculture Organisation (FAO 2007) observes that the crop failures have been a result of early termination of the rains in most seasons or low rainfalls in Zimbabwe, especially in semi-arid areas. The sudden decline in yield and output at farm level has led to a shortfall in agricultural production to meet annual food requirements for the general populace. In 2002, Zimbabwe experienced the largest deficit in its food production since 1980 (Manyeruke, Hamauswa & Mhandara 2013; Mupindu 2016; Nyahunda & Tirivangasi 2019). This was a blow that created severe food shortages in both urban and rural areas. The food shortages deteriorated into a famine and a humanitarian disaster. The Zimbabwe Vulnerability Assessment Committee (ZimVAC 2002) posits that the cereal deficit in the April 2002 to March 2003 marketing year was estimated at 1.65 million tonnes. The World Food Programme (WFP 2002) points out that, of the 6 700 000 people requiring food aid, 5 900 000 were in semi-arid areas of Zimbabwe and 800 000 in urban areas. Seventy per cent of the rural population was at risk of famine-induced starvation. The scale of the food aid was unprecedented in the history of Zimbabwe.

The agricultural sector is the backbone of the Zimbabwean economy, contributing 15% – 20% to the GDP, 40% to exports and 60% of the raw materials used by the domestic manufacturing industry (ZUNDAF 2011:9). In Zimbabwe, the past decade has seen an increase in food and nutrition insecurity at household and national levels emanating from reduced productivity and production of the main crops, partly as a result of climate change and other socio-political events that were unfolding in the country. All this is attributed to the country’s lack of a comprehensive agricultural policy model. Recent studies using global circulation models have shown that from the current period up to the year 2080, Zimbabwe will face a general decrease in reliability and predictability of rainfall patterns while temperatures are expected to rise by 2°C (Bohle, Downing & Watts 1994:47). Such a change has a serious impact on the country’s food security, thus causing the need for contingency measures to be put in place. It is now universally agreed that climate change and climate variability are among the greatest challenges facing mankind in the 21st century. In Zimbabwe recent concerns have been raised that no one is taking the responsibility to advise farmers on when to plant, what to plant and how to plant in line with the changing climate that has become a reality.

### Operationalisation of variables

#### Climate change

Burroughs (2002) opines that climate change is a phenomenon that is born out of the carbon dioxide that is released on Earth and gets trapped in the ozone layer, which protects harmful ultraviolet rays from hitting the Earth’s surface. In support of this view, Reid et al. (2009) indicate that these gases trapped in the ozone layer give rise to global warming, also called ‘climate change’, which is responsible for altering weather conditions. The alteration in weather conditions results in droughts, heat waves, floods and unpredictable weather patterns, intense storms, and decreased agricultural productivity and rising food insecurity. Meanwhile, the Intergovernmental Panel on Climate Change (IPCC 2007) argues that climate change is a change in the state of the climate that can be identified by using statistical tests through observing changes in the mean and the variability of its properties, and that persists for an extended period, typically decades or longer.

#### Food security

Food security is when all people, at all times, have physical and economic access to sufficient, safe and nutritious food to meet their dietary needs and food preferences for healthy and active life (FAO 1996). The definition of ‘food security’ was further broadened by the advent of Amartya Sen’s book *Poverty and Famines in 1981*. Sen’s book dwells on the point that the starving are often denied access to food rather than suffering because food is unavailable and by so doing birthed the idea of entitlement to food. Sen (1982) posits that starvation is the characteristic of some people not having food to eat; it is not the characteristic of there being not enough food to eat. The idea was to make a paradigm shift in the concept of food security out of the essentially agricultural domain and place it in a wider context of poverty and lack of development. This culminated in the Food and Agriculture Organisation adding the component of access to those of production and price stability.

According to the Food and Agriculture Organization (FAO 1983), ‘… the ultimate objective of world food security should be to ensure that all people at all times have both physical and economic access to the basic food they need’. Food security should have three specific aims, namely ensuring production of adequate food supplies, maximising stability in the flow of supplies and securing access to available supplies on the part of those who need them. However, it is highly debatable whether access, although it is a social factor in food security, is sufficient alone or should be accompanied by stability. More so, food insecurity could be grouped into two categories, that is either chronic or transitory, with the former indicating a situation where the lack of food is a permanent feature and the latter represents a temporary shortage. Chronic food insecurity can be best described as when the risk of famine is high and that to establish food security that phenomenon must be dealt with and uprooted, giving rise to the idea of access of all people at all times to enough food for an active healthy life.

#### Adaptation

Adaptation is widely recognised as a vital component of any policy response to climate change. Rural farmers, especially women, whose livelihoods depend on the use of natural resources are likely to bear the brunt of the adverse impacts of climate change. Adaptation is oriented towards longer-term livelihood security, a continuous process, sustained results and efficient use of resources; it involves planning, combines old and new strategies, and knowledge is focused on funding alternatives (Care 2007). Adaptation can greatly reduce vulnerability to climate change by making rural communities better able to adjust to climate change and variability, moderating potential damages and helping them cope (Bryan et al. 2009). Notably, adaptive capacity is a function of many factors including availability and distribution of resources, social and human capital, access to risk spreading and ability to process information (Corfee-Morlot & Hohne 2003). The Southern African region has the highest prevalence of food insecurity as a result of climate change, and adaptation to climate change varies with local conditions. The IPCC (2001) defines ‘adaptation’ as adjustments in ecological, social or economic systems in response to actual or expected climatic stimuli and their effects or impacts. This term refers to changes in processes, practices and structures to moderate potential damages or to benefit from opportunities associated with climate change.

### Theoretical framework

The theory underpinning this study is Sen’s entitlement theory, whose theorising was on the analysis of famines. Entitlements have been defined by Sen (1985:497) as ‘the set of alternative commodity bundles that a person can command in a society using the totality of rights and opportunities that he or she faces’. Sen (1982:166) concludes poverty and famines with this famous observation: ‘The law stands between food availability and food entitlement’. This portrays the law as a barrier that restricts people from getting food from shops, farms and neighbours, to mention but a few. If people try to get food from those who have it without their consent, it might be regarded as a serious crime. In other words, it may mean that even though food is available it might not be easy to access, leading to hunger and starvation. Edkins (1996:550) propounds that, according to ‘Sen’s framework, people destitute by famine are not entitled to food; instead they are entitled to starve’. A person’s entitlement set is the full range of goods and services that he or she can acquire by converting his or her endowments, that is, assets and resources, including labour power.

Furthermore, Amartya Sen’s progressive research on food security changed the course of research into food security by bringing the aspect of accessibility of food to the forefront. Baiphethi and Jacobs (2009) observe that previously researchers had focused their research on food production and availability. Sen (1982), in what he termed ‘entitlement’, pointed out that there are factors such as access to land, credit and support services that can result in individuals in society failing to access food. Sen’s work is more appropriate in the African context, where developing countries are evolving. As a result of factors such as globalisation and migration, the population has moved from being mostly Zimbabwean subsistence farmers to being consumers.

## Methodology

### Study area

**FIGURE 1:**
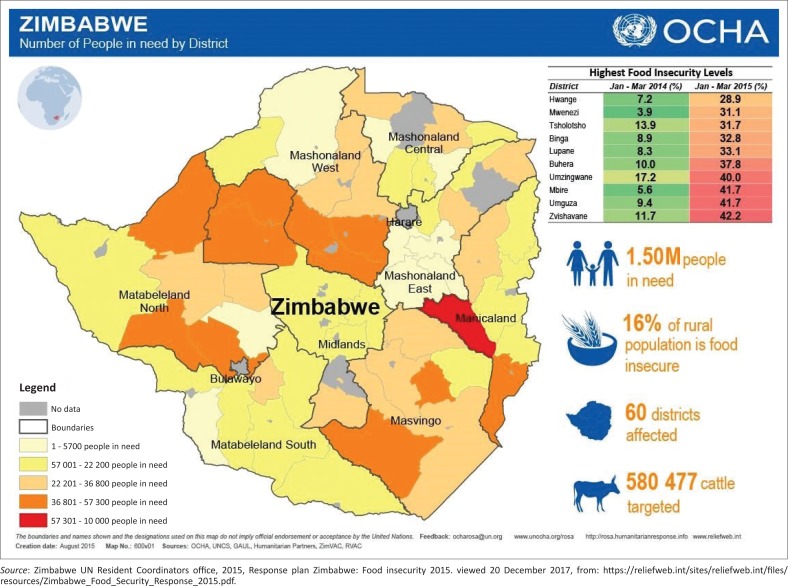
Food insecurity levels per province.

**FIGURE 2:**
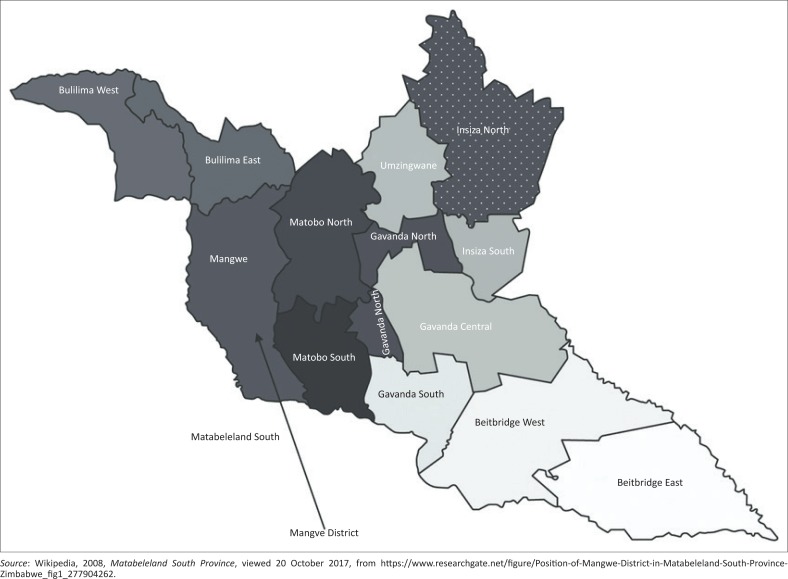
Location of Mangwe District in Matabeleland South Province, Zimbabwe.

Mangwe is a constituency located in Matabeleland South as clearly shown in Figures 1 and 2. It is characterised by chronic food shortages (ZESN 2010 Report). The constituency has a lot of wildlife and unexploited natural resources such as natural gas and timber. However, according to the 2003 Poverty Assessment Study Survey Summary report, the whole district has high poverty rates of 64%, and most of the households are female headed (ZESN 2010 Report). There is also a low electrification rate – 93% of households are not electrified. Moreover, 20% – 30% of households are food-insecure; however, it is one of the constituencies with over 50% food security (ZESN 2010 Report).

### Research design and sampling

The researcher adopted an exploratory research design for this secondary research. Burns and Grove (1997:38) define exploratory research as research conducted to gain new insights, discover new ideas and learn the truth about something. A purely qualitative research design and a constructivist grounded theory approach was used in this study. In other words, the study used an exploratory design that was sequentially timed. As the research evolved, the researchers constructed meaning from the data collected. The respondents were selected through purposive sampling to ensure that only respondents who had information on food insecurity were selected. Expert and critical case sampling are examples of purposive sampling used in this study. Regarding expert sampling, the study focused on experts in the field in the District Food and Nutrition Committee, whereas for critical case sampling the study focused on those wards that had the highest cases of food-insecure people.

Traditional leaders and communal farmers were also among the respondents. Moreover, key informant interviews were also conducted with development partners from both government and non-state actors specialising in small grains. Participants included six officers from the Department of Agricultural, Technical and Extension Services (AGRITEX) office, two officers from the district administrator’s office, two from the Rural District Council, two from the Department of Agricultural and Rural Development Authority and 60 farmers. These respondents were chosen and targeted particularly because AGRITEX officers, for example, are directly linked to the coordination of small grains in the Mangwe District. The 60 farmers selected were the ones responsible for the production of small grains or choosing not to produce; hence their views were of paramount importance. Key informant interviews were advantageous because the officers provided saturated information about the subject under interrogation. It allowed divergent views from various stakeholders, from which a central story line was deduced.

### Data collection and data analysis

The researchers triangulated the data collection tools. Triangulation is defined as a process of using multiple perceptions to clarify meaning, verifying the repeatability of an observation or interpretation (Denzin & Lincoln 2008:133). The research used qualitative methods of collecting data, which is focused group discussions, key informant interviews and secondary data. Qualitative methods were suitable to explore the impact of small grains as an adaptive strategy to climate change. Focus group discussions were also instrumental in gathering data at district level, where farmers in the district with different farm sizes were involved.

Analysis of the interview transcripts and field notes was done in themes using NVIVO software (Charmaz 2006). Data analysis was conducted at the same time as the data collection in a process that was iterative and comparative of evolving data. This included open coding, selective coding and axial coding in all data analysis phases. Further, data was analysed using NVIVO software, which squarely fits qualitative research because analysis brings out themes.

### Ethical considerations

Approval for the study was obtained from the University of KwaZulu-Natal (reference number: HSS/0185/017D).

## Findings and discussions

### Small grains for adaptation to climate change

Small grains have been noted as staple food grains in many semi-arid and tropical areas of the world, particularly in sub-Saharan Africa, because of their good adaptation to harsh environments and their good yields of production. Bang and Sitango (2003:45) note that small grains are generally the most drought-tolerant cereal grain crops, requiring little input during growth; and with increasing world populations and decreasing water supplies, they represent important crops for future human use. Small grains have the potential to contribute towards the food security of many of the world’s poorest and most food-insecure agro-ecological zones. This can be achieved through increasing production and productivity of these crops in such agro-ecological zones. These conclusions align with those of Bang and Sitango (2003), that small grains have the potential to improve household security in semi-arid regions because of their adaptability to such environments. Despite this, research on these crops has been lagging behind in Africa because they suffer something of an image problem, and there often tends to be a preference for maize as the premier crop.

Small grains like sorghum and finger millet have proved to have a high percentage of availability. For instance, finger millet when properly dried can be stored for a period of up to 5 years or more. This promotes its availability and therefore strengthens the food security status in the district. Dube (2008) posits that some of the advantages of small grains like sorghum and millet over maize include the following: a smaller amount of flour is needed to cook the main meal compared to maize, and a meal cooked from small grains satisfies hunger for a longer period and gives more energy (which is especially important for persons who do heavy manual labour like farmers).

Maize is not a drought-tolerant crop; hence, when it is planted in dry areas, it is highly affected by harsh environmental conditions, which affect the yield. The limited lifespan of maize also reduces its availability, because it can be stored for not more than 2 years. This reduced lifespan of maize leverages the production of small grains in drought-prone areas, as they can be stored for more than 5 years. Most households in Mangwe, especially those that are regarded as poor, are likely to have their maize grain stock run out immediately in less than a year. The reason could be that maize needs special attention ranging from fertiliser application to weeding. However, this is totally different from small grains, which do not require many inputs such as fertilisers and pesticides.

According to a farmer in Madabe in Mangwe:

‘The area of Madabe is very hot, and rainfall sometimes is very low. However, this year rainfall was too much and all the maize crops [became] submerged in water. Had it been the fact that we were given fertiliser in time, we were going to realise a bumper harvest. But for those who planted small grains they managed to get something.’ (Male, farmer, age 60)

The researcher noticed several responses similar to this, which showed that Mangwe is no longer suitable for maize production. It can be deduced from the responses mentioned earlier that in the Mangwe District small grains have a positive impact on making the population food secure as compared to when maize is grown. This evidence is justified by the trends showing that Zimbabwe is increasingly becoming food-insecure because of its reliance on maize. However, evidence reveals that, more than before, there has been an increase in small seed production, as highlighted in Figure 4.

**FIGURE 3:**
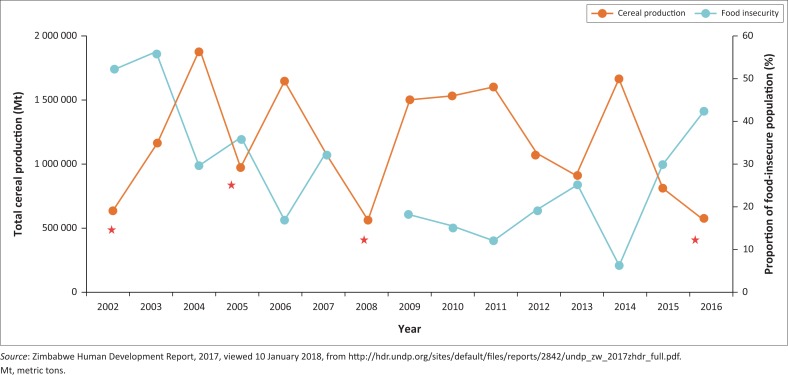
Trends in cereal production and food insecurity, 2002–2016.

**FIGURE 4:**
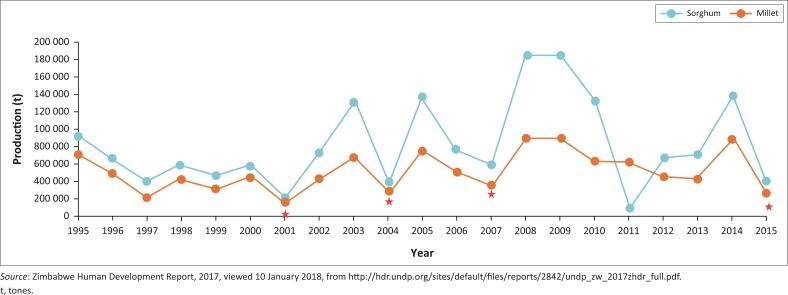
National small grain production, 1995–2015.

The trends in Figure 3 reveal that Zimbabwe is becoming increasingly food-insecure. Maize stock deficits continue to widen. This is because of the non-resistant nature of maize to climate change, which demands a shift to small grain production. Figure 4 shows that the production of small grains has been on the rise since 1995, and this calls for the adoption of crops such as sorghum and millet, as they are more drought resistant. This study has revealed that farmers who grew small grains in Mangwe managed to reap something to sustain themselves.

The trends revealed in Figure 4 clearly show that production of sorghum and finger millet was low during the 1990s; however, with the growing impacts of climate change on maize, drought resistance has been gaining the interest of the farmers. This evidence reveals that small grains can be successfully used as an adaption strategy to alleviate food shortages, strengthen grain reserves and build resilience.

### Consumption

In the Mangwe District, emphasis is on small grains to ensure that food accessibility is promoted and guaranteed. Accessibility has been realised and increased through the production of finger millet specifically for those farmers who are growing pearl and finger millet. It is imperative to note that those households that have embarked on small grain production have now realised food accessibility. Further, both finger millet and sorghum contributed immensely to food accessibility as they are readily available in the district. Sen’s entitlement theory asserts that the law stands between food availability and food entitlement. However, it can be argued that, even though the production of finger millet and sorghum was substantial in the Mangwe District, some parts of Mangwe were not able to access the food from the Grain Marketing Board because they did not have enough money to purchase the grain. The theory further asserts that famines make people who are not entitled to food rather entitled to starve. When people fail to access food from either the shops or the Grain Marketing Board, this makes the populace starve. Sen (1982) emphasises food accessibility over availability. Small grains contributed a great deal to food availability at the expense of food accessibility. Sen equated entitlements with factors such as access to land, credit and support services. If these fail, an individual can fail to access food. This deep analysis was reflected in Mangwe, whereby those farmers who did not have access to arable land, credit and support services from stakeholders could not produce enough for family consumption.

**FIGURE 5:**
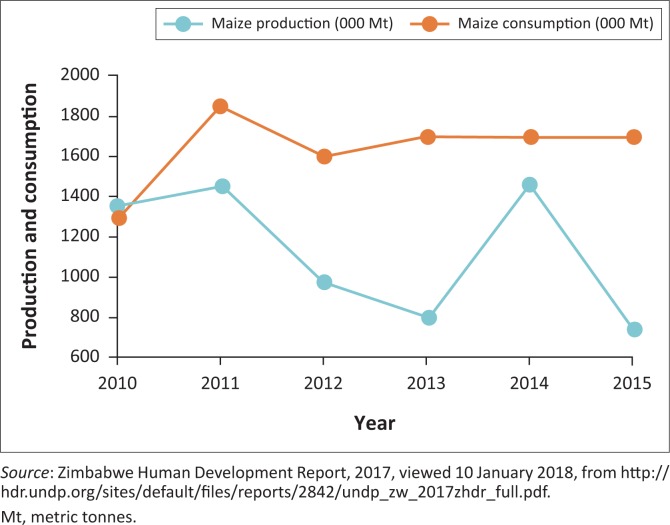
Maize production and consumption trends, 2010–2015.

According to Ndlovu (2011), drought resistant crops such sorghum, pearl millet, cowpeas and groundnuts have become extremely important to the local community This is because of the fact that they act as both food and cash crops, which enables smallholder farmers to adapt to climate change and variability and attain sustainable livelihoods. Small grains in Mangwe have been used for a long time for beer brewing. The study revealed that red sorghum is being used extensively by beer brewing companies such as Ingwebu in Bulawayo City. This gives farmers money to earn a living after selling the red sorghum both to the Ingwebu Company and the Grain Marketing Board. The study concludes that traditional beer is therefore an important source of cash income in the semi-arid smallholder sector. The results from literature reveal that most people in Zimbabwe have a higher consumption of maize, in accordance with the trends shown in Figure 5. However, these trends clearly show that maize production levels are way behind consumption levels, which renders most communities food-insecure.

Murisa and Mujeyi (2015) note that maize stock deficits continue to widen, and the country is being forced to rely on food aid. The trends here, which continually show how Zimbabwe is increasingly becoming food-insecure, point to the need for the adoption of small grains by communities like Mangwe and others affected by climate change.

### Challenges in the production of small grains

The FAO (2000) posits that production of small grains continues to suffer because it offers low yields. The FAO (2010) further propounds that the low yields of small grains have acted as a major impediment for communal farmers in Zimbabwe’s semi-arid regions to expand and adopt production of small grains on a large scale compared to maize. The lower productivity of small grains causes farmers to neglect the production of small grains in Mangwe.

#### Lack of government support

Rukuni (1994) argues that lack of government support in Zimbabwe for production, processing and use of crops that are tolerant to drought has resulted in people in the drier areas changing their tastes from small grains to maize. The only research station in Matabeleland is in Matopo, and most researchers are isolated from the farmers. Most research is initiated on research stations; not enough takes place on farms. More emphasis should be on researching problems on farms using a multidisciplinary approach whereby teams are actively engaged to analyse indigenous technologies and current farmer practices.

#### The problem of quelea birds and small grains

The quelea birds in the Mangwe District present a challenge to farmers as they eat the grains, which reduces the yield. Research has shown that the ever-increasing labour costs that are involved in the production of small grains affect most farm operations, from land preparations, weeding, bird scaring to harvesting and grain processing (Barrett & Maxwell 2005). Further, the reduced labour involved in the production of maize compared to the extensive effort needed in the production of small grains gives maize the upper hand and explains why maize became widely accepted, even in Zimbabwe’s semi-arid regions and even after independence.

#### Lack of markets

One chief concern, among others, is the problem of limited marketing opportunities, as cited by most respondents in the district. Despite a lot of publishing and research that has been conducted for small grains, there is still a lack of markets. Most respondents were concerned about the absence of a ready market for small grains. If farmers had buyers who would buy their small grains at a lucrative price, it could transform the lives of farmers in the Mangwe District. In Mangwe farmers cannot rely on the Grain Marketing Board to sell their small grains as their prices are not sustainable for growth. It is the farmers’ expectation that production and post-harvest costs be met by the profit realised after selling, but unfortunately the demand for small grains is still minimal in Zimbabwe.

#### Labour

Regarding barriers to the adoption of small grains, Scoones (1998) noted that labour is required for both cultivation and processing.

One of the officers from the Ministry of Agriculture indicated that:

‘… the production [of] small grains, whatever type, is very labour intensive and many people find it difficult to rise to the demands required in harvesting small grains.’ (AGRITEX officer, male, age 42)

In Mangwe many people agreed that weeding is one of the most demanding aspects, requiring people with advanced skills because most of the weeds look similar to finger millet. It is therefore a hard task if a farmer engages a person who is not able to differentiate a weed from finger millet. One may end up destroying finger millet while attempting to target the weed. This leads to reduced yields because the desired crop is destroyed. In Mangwe most women complained about the increased labour that is involved, from sowing to harvesting. A number of women noted that, although small grains are a contributing factor in achieving food security, the labour and effort needed to produce them is just unbearable. They pointed out that the crop has small grains that prove to be laborious, especially during harvesting time. Because the seeds are small, it takes skill and much effort to mill finger millet, especially by hand (Svodziwa 2015). Hammer mills have to be fitted with very fine screens and run at high speed, but the National Research Council (1996) reported the development of a special mill for millet (Svodziwa 2015). Subsequently weeding is becoming a major problem, especially during the early stage of crops, and this is labour intensive. The difficulty in weeding is complicated by wild relatives of the crop (e.g. *Eleusine indica*) that look like finger millet at the time of weeding (Svodziwa 2015). The problem of seed size carries over into processing (Carney 1999).

## Conclusion

The growing of small grains in drought-prone areas has proven fruitful when compared to maize production. The study found that the price for small grains at the Grain Marketing Board is very low and this chases many farmers away from selling their farm produce there. In that regard, the government of Zimbabwe through the Ministry of Agriculture should come up with a clearly articulated policy that promotes the production of small grains. This can be achieved through offering competitive prices. Intra-rural markets can also be established so that farmers do not have to travel long distances to the market. The government should assist financially handicapped farmers such as those in semi-arid areas like Mangwe. The presidential input scheme should pay special attention to small grains that do particularly well in the Mangwe District. Small grains have been noted by research experts to have a better yield in drought-prone areas and are considered to have better nutritional content than maize, which is viewed as an undependable crop in these agricultural ecological zones. Research revealed that small grain production is one of the major dependable ways of addressing food shortages in Mangwe. In a region such as Mangwe, where agriculture is dominant as a source of many people’s livelihoods, swift response to climate change effects should be treated as a priority.
